# Oxygen saturation measurements using novel diffused reflectance with hyperspectral imaging: Towards facile COVID-19 diagnosis

**DOI:** 10.1007/s11082-022-03658-z

**Published:** 2022-05-08

**Authors:** Yasser H. El-Sharkawy, Mohamed Hisham Aref, Sherif Elbasuney, Sara M. Radwan, Gharieb S. El-Sayyad

**Affiliations:** 1 Head of Biomedical Engineering Department, Military Technical College, Egyptian Armed Forces, Cairo, Egypt; 2 Biomedical Engineering Department, Military Technical College, Egyptian Armed Forces, Cairo, Egypt; 3 Head of Nanotechnology Research Center, Military Technical College, Egyptian Armed Forces, Cairo, Egypt; 4 Biochemistry Department, Faculty of Pharmacy, Ain Shams University, Cairo, Egypt; 5 Microbiology and Immunology Department, Faculty of Pharmacy, Galala University, New Galala city, Suez, Egypt; 6 Chemical Engineering Department, Military Technical College, Egyptian Armed Forces, Cairo, Egypt

**Keywords:** Vein viewer, Hyperspectral imaging, Oxygen concentration, K-means clustering algorithm, Tissue characterization

## Abstract

Oxygen saturation level plays a vital role in screening, diagnosis, and therapeutic assessment of disease’s assortment. There is an urgent need to design and implement early detection devices and applications for the COVID-19 pandemic; this study reports on the development of customized, highly sensitive, non-invasive, non-contact diffused reflectance system coupled with hyperspectral imaging for mapping subcutaneous blood circulation depending on its oxygen saturation level. The forearm of 15 healthy adult male volunteers with age range of (20–38 years) were illuminated via a polychromatic light source of a spectrum range 400–980 nm. Each patient had been scanned five times to calculate the mean spectroscopic reflectance images using hyperspectral camera. The customized signal processing algorithm includes normalization and moving average filter for noise removal. Afterward, employing K-means clustering for image segmentation to assess the accuracy of blood oxygen saturation (SpO_2_) levels. The reliability of the developed diffused reflectance system was verified with the ground truth technique, a standard pulse oximeter. Non-invasive, non-contact diffused reflectance spectrum demonstrated maximum signal variation at 610 nm according to SpO_2_ level. Statistical analysis (mean, standard deviation) of diffused reflectance hyperspectral images at 610 nm offered precise calibrated measurements to the standard pulse oximeter. Diffused reflectance associated with hyperspectral imaging is a prospective technique to assist with phlebotomy and vascular approach. Additionally, it could permit future surgical or pharmacological intercessions that titrate or limit ischemic injury continuously. Furthermore, this technique could offer a fast reliable indication of SpO_2_ levels for COVID-19 diagnosis.

## Introduction

The level of tissue oxygenation is an essential determinant for diagnosis and treatment follow-up of numerous diseases (Miclos et al. 2015). Arterial blood oxygen saturation (SaO_2_) is considered as the fraction of oxygen-saturated hemoglobin proportionate to total hemoglobin (Chernobay et al. 2020). The human body necessitates and controls a very precise balance of oxygen in the blood (van Gastel et al. 2016b). Normal SaO_2_ levels in humans are 95–100%, while below 90 percent is considered low and called hypoxemia (Qadir &Naeem 2019). Several factors including the local, and pulmonary blood circulations may affect blood oxygen saturation (Collins et al. 2015). Thus, measurement of oxygen saturation is critical for monitoring pulmonary and cardiovascular functions as well as evaluating peripheral vascular diseases such as the case in diabetic foot ulcers (Miclos et al. 2015; Orchard and Strandness 1993). Regarding the COVID-19 pandemic, many patients are suffering from low oxygen levels (Abd Elkodous et al. 2020). This can be an early warning sign for urgent medical care. Therefore, to control this global threat, non-contact, COVID-19 testing capabilities for oxygen saturation measurement is an urgent need. This approach could offer a novel system for safe airport travel for arriving and departing passengers (Tzivakou 2020). Several techniques including blood gas analysis (Davis et al. 2013), transcutaneous oxygen measurement (Rithalia 1991), near-infrared spectroscopy (Hyttel-Sorensen et al. 2011), and magnetic resonance imaging (Christen et al. 2014), have been recently developed for tissue oxygenation measurement. Additionally, the use of a pulse oximeter has become a gold standard in measuring SpO_2_ (Ralston et al. 1991). Unfortunately, pulse oximeter sensor requires direct contact with the skin, which causes skin irritation and discomfort (Jubran 2015). However, the different limitations of previous techniques have highlighted the need for the development of rapid, non-invasive, remote monitoring SpO_2_ technique (van Gastel et al. 2016b). The convenience of remote systems for monitoring oxygen saturation levels has been an increasingly studied topic, with growing evidence of its success (Appelboom et al. 2014). Visible light can penetrate the skin; its interaction with cellular tissue can provide a signature about SpO_2_ level (O'doherty et al. 2007). Consequently diffused light reflectance with hyperspectral imaging (HSI) technique could experience high capability to distinguish the full-color spectrum in each pixel (Panasyuk et al. 2007). This novel technique can offer spectral information as well as 2D spatial images, thus offering great potential for non-invasive disease diagnosis, surgical assistance. Furthermore, it could provide a quick indication of COVID-19 pandemic diseases (Hosking et al. 2019). The light delivered to biological tissue undergoes multiple scattering and/or absorption as it disseminates through the tissue. Consequently, the reflected, fluorescent and transmitted light captured by HSI delivers quantitative diagnostic information (Lu and Fei 2014). Image analysis and computational power enable processing the large amount of data included in hyperspectral images (Miclos et al. 2015). Novel non-invasive diffused reflectance with HSI technique can provide the mapping of both oxy- and deoxy-hemoglobin distribution (Chin et al. 2012).

The commercial pulse oximeter had been known as the ground truth in measuring the blood oxygen saturation (SPO_2_) in the last 70 years ago, where it is basic phenomena relies on two different LED (red and near infra-red (NIR)) as a light source. Although, the red at wavelength range 660 nm and NIR at wavelength range 940 nm. Then, the light absorption fluctuates with respect to the oxygen saturation in the blood vessels, through these variations scientist were capable to calculate the SPO_2_ (Aoyagi 2003; Wukitsch et al. 1988; Zonios et al. 2004). However, it had several limitation such as: good contact with the device probe with the patient finger, fluctuation of the devices sensitivity, and the importance for good blood flow reaching the extremities for the measurement possibility (Aref et al. 2021).

Therefore, in this article, we proposed the development of a customized optical diffused reflectance with HSI to assess the levels of SPO_2_ in minimum time and non-contact method. The system outcomes were evaluated with the gold standard technique, a standard pulse oximeter, and good agreement between the developed non-contacts diffused reflectance technique and pulse oximeter measurements.

## Experimental work

### Subjects

For this investigation, 15 healthy adult male volunteers with age range of (20–38 years) were recruited. An informed written consent was obtained from all the study participants. The study was approved by the Ethical Committee of Research, in accordance with the Declaration of Helsinki, Cairo University, Egypt (No: P.T.REC/009/003144). All volunteers read and signed a two copies of consent form prior the start of data collection. The exclusion criteria included those suffering from any respiratory, cardiac, and peripheral vascular disease.

### Principle and procedure

The levels of oxyhemoglobin (HbO_2_) and deoxyhemoglobin (Hb) can characterize blood oxygenation with good accuracy and can be employed as monitoring parameters for pulmonary and cardiovascular functions (Miclos et al. 2015). The pulse oximetry is the gold standard for non-invasive SpO_2_ measurements in blood since 1930s (Velavan and Meyer 2020). However, the HbO_2_ and Hb molecular configurations lead to different electromagnetic absorption and therefore different emission of light. Oximeters operation is based on the principle of different absorption and light emission (Blaisdell et al. 2000).

The conventional oximeter utilized two different LED (Red at wavelength 660 nm and NIR at wavelength 940 nm) absorption of light at these wavelengths differs significantly between blood loaded with oxygen HbO_2_ and blood lacking oxygen Hb. The HbO_2_ absorbs more infrared light and allows more red light to pass through, while Hb allows more infrared light to pass through and absorbs more red light (Velavan and Meyer 2020).

The main principle of optical imaging for blood oxygen saturation (SpO_2_) includes three different forms of light interaction with the tissue (specular reflection/diffused reflection/scattering reflection) (Pascarella et al. 2020). Diffused reflection is the result of light reflection from irregular surfaces (Khonina and Golub 2012, O'Sullivan et al. 2012).

**Fig. 1:**
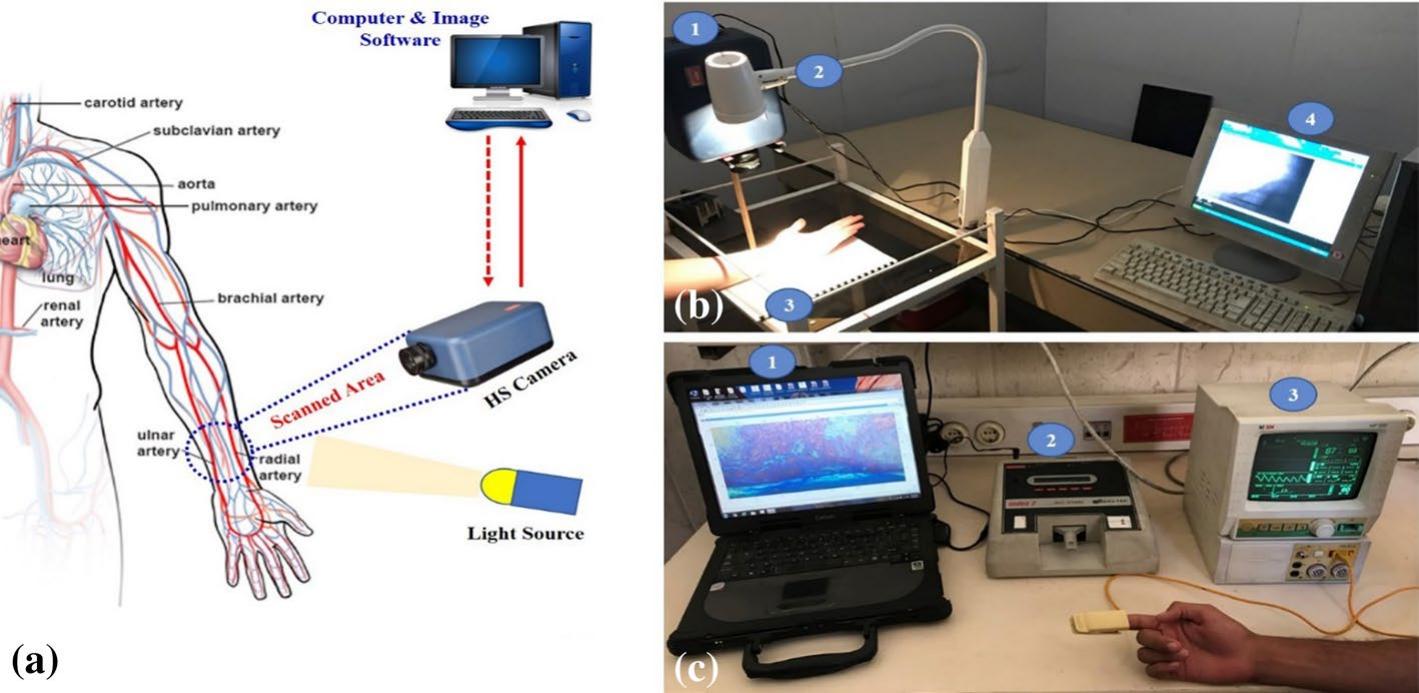
The proposed Non-contact diffused reflectance system for blood oxygen concentration; **a** The schematic diagram for the proposed system highlighting the blood circulation of the ROI in the forearm (subclavian artery and subclavian vein) to measure the oxygen saturation without contact from the spectral images; **b** The actual setup, where (1) the hyperspectral camera (Surface Optics, SOC710, USA), (2) Polychromatic source light (Derungs, 400–100 nm, Germany), (3) Investigated ROI of the subject Forearm, (4) The computer and software analysis;** c** Validation of the measurement with a standard commercial pulse oximeter, where (1) the computer and software analysis, (2) Calibration equipment (Biotek,index2, USA), (3) Patient Monitor (MEK, MP500, Korea)

Customized optical imaging system was developed; where the volunteer hand was illuminated using white hydrogen lamp source (400–900 nm). The key element to acquire the necessary cube images is the Hyperspectral Camera (Surface Optics, SOC710, USA) with spectral range (400–000 nm), 4.68 nm spectral resolution, 128 spectral bands. The camera combined with lens (Schneider, 1.9/35 CCTV, 400–1000 nm, Germany). The optical setup was aligned and fixed during all the experimental trial; where the distance between the focal point and the investigated sample (forearm of the participants) was kept steady at 20 cm. Each patient had been scanned five times and calculating the outcome mean, then validating the captured data of the oxygenated arteries and de-oxygenated veins with respect to the conventional pulse oximeter measurements; two different devices were employed including (Patient Monitor, MEK, MP500, Korea) and (Pulse finger, Contec, CMS50D, China). Figure 1 is a schematic of customized non-contact diffused reflectance optical imaging system to standard pulse oximeter measurements.

For every volunteer, 2-cube images were consecutively captured for the same skin spot. Consequently, HS camera was employed for all the subjects. RGB-IR image segmentation was applied for the region of the interest (ROI) to highlight the oxygenated arteries and de-oxygenated veins. Afterwards, the captured diffused reflectance signals for various illuminating wavelengths were investigated; the optimum signature for SpO_2_ level was recorded at 610 nm. Moving average filter was employed for noise removal and image enhancement. Finally, the K-means clustering on the optimum wavelength images (610 nm) was applied to delineate the oxygenated arteries and the de-oxygenated veins, respectively.

### Designed digital signal and image processing algorithm

Customized algorithm was developed in an attempt to measure the optical properties (reflection and the diffused reflectance) for the investigated subjects. For HSI calibration and advanced image procurement for data normalization, two different captured images for a highly reflected (white cube) and total opaque (dark cube) were employed (Md Noor et al. 2017). The basic purpose is to eliminate artifacts and noise impacts on the investigated sample tissue, as represented in Eq. (1):

1 $$\mathop {\mathbf{R}}\limits_{{\mathbf{,}}} \left( \vartheta \right) = \frac{{\tilde{I}_{{\varvec{m}}} \left( {{\varvec{\Phi}}} \right) - \tilde{I}_{{\varvec{d}}} \left( {{\varvec{\Phi}}} \right)}}{{\tilde{I}_{{\varvec{w}}} \left( {{\varvec{\Phi}}} \right) - \tilde{I}_{{\varvec{d}}} \left( {{\varvec{\Phi}}} \right)}} \times 100\%$$

where, $\mathop {\text{R}}\limits_{,} \left( \vartheta \right)$ is the reflectance of the subject image, $\tilde{I}_{m} \left( \Phi \right)$ is the acquired image,$\tilde{I}_{d} \left( \Phi \right)$ is the dark cube image, and $\tilde{I}_{w} \left( \Phi \right)$ is the white cube image.

Acquired images were normalized to eliminate the interfering spectral signature from polychromatic light source. Consequently, moving average filter (K = 10) was applied. Finally, the K-means image segmentation (K = 10) was employed. Further details about employed equations have been provided in previous studies (Aboughaleb et al. 2020; Aref 2020). Every collected image by HS camera was exploited to calculate the light attenuation. Finally, the contour delineation of the oxygenated arteries and the de-oxygenated veins was overlaid on the images of 610 nm wavelength.

## Results and discussions

### Optimization of diffused reflectance spectrum

**Fig. 2:**
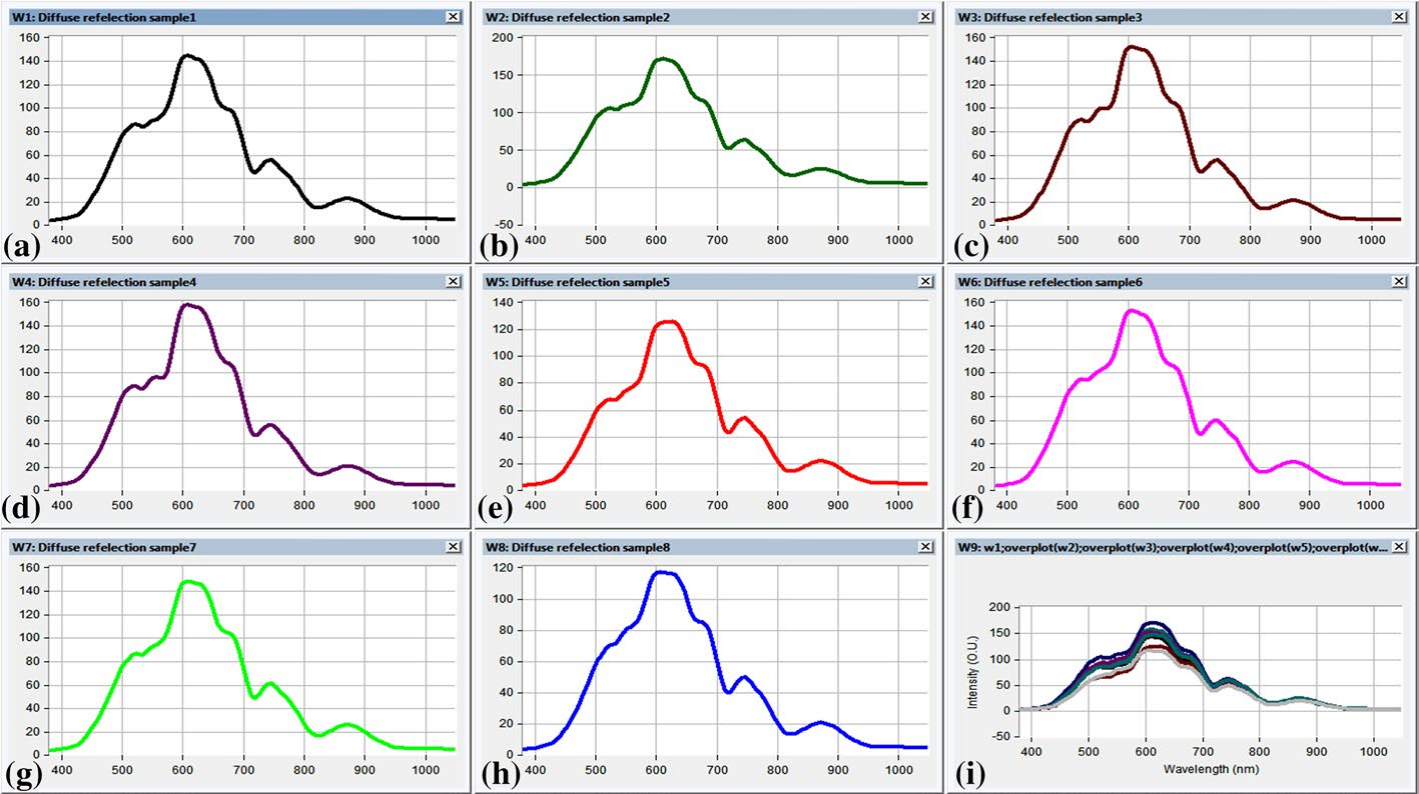
The measured diffused reflectance signatures of the volunteer`s forearm exploiting the hyperspectral camera under illumination with the polychromatic light source with range 400–900 nm for the eight adult volunteers, **a** The measured diffused reflectance signatures of the volunteer`s forearm Volunteer#1, **b** The measured diffused reflectance signatures of the volunteer`s forearm Volunteer#2, **c** The measured diffused reflectance signatures of the volunteer`s forearm Volunteer#3, **d** The measured diffused reflectance signatures of the volunteer`s forearm Volunteer#4, **e** The measured diffused reflectance signatures of the volunteer`s forearm Volunteer#5, **f** The measured diffused reflectance signatures of the volunteer`s forearm Volunteer#6, **g** The measured diffused reflectance signatures of the volunteer`s forearm Volunteer#7, **h** The measured diffused reflectance signatures of the volunteer`s forearm Volunteer#8, **i** The compound of the measured diffused reflectance signatures for the eight adult volunteers in the investigation

The variance in diffuse reflectance intensity due to change in oxygen blood level was measured using hyperspectral camera (Aref et al. 2021). Total fifteen volunteers were classified into two groups. Group1 of eight volunteers were subjected to this study; each volunteer forehand was illuminated with white hydrogen lamp source (400–900 nm). The recorded diffused reflectance spectrum signals for adult eight volunteers are represented in Fig. 2.

**Fig. 3:**
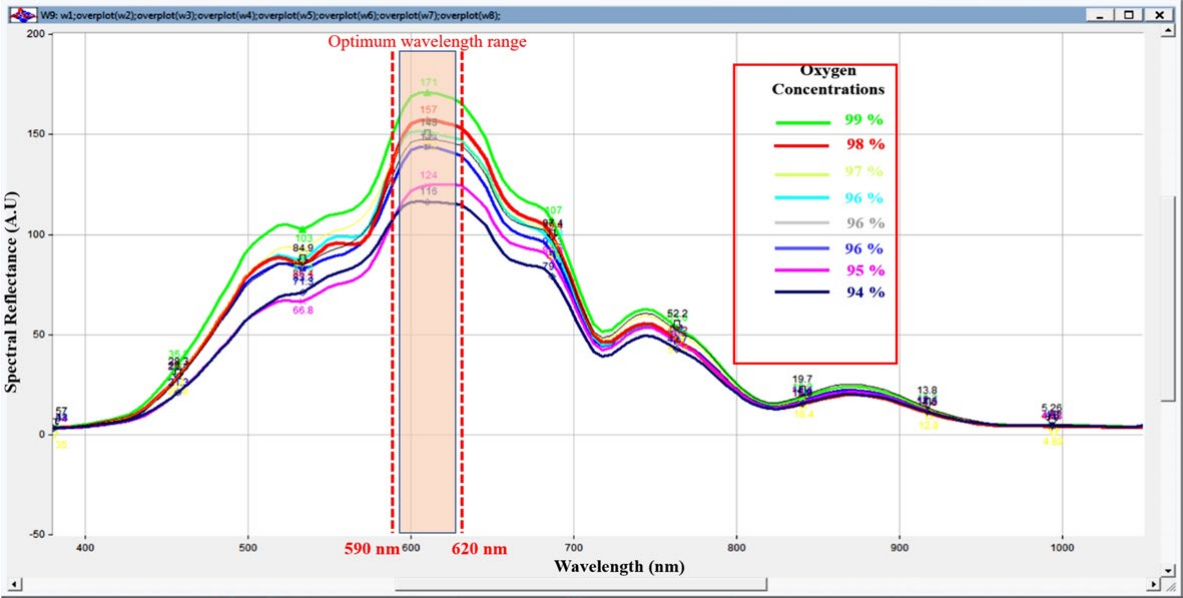
The measured diffused reflectance signatures of the volunteer`s forearm highlighting the optimum wavelength range with maximum reflection at range (590–620 nm) with respect to the actual SPO2 measurements by the commercial pulse oximeter of the selected investigated eight volunteers

It is obvious that there is a variation in diffused reflectance spectrum signal; this variation could be function of oxygen blood concentration. The maximum change in oxygenated and de-oxygenated blood level could occur at optimum diffused reflected wavelength. Maximum variation of diffused reflectance spectrum occurred at 610 nm. The oxygen blood concentration for investigated eight volunteers was measured using commercial pulse oximeter, in an attempt to evaluate the capability of diffused reflectance system to measure the oxygen level (Kyriacou et al. 2009). Good agreement between the developed non-invasive, non-contacts diffused reflectance system with pulse oximeter results. It is apparent that maximum deviation in diffused reflectance signal (correlated to change in oxygen blood level) was recorded at 610 nm, as shown in Fig. 3.

**Fig. 4:**
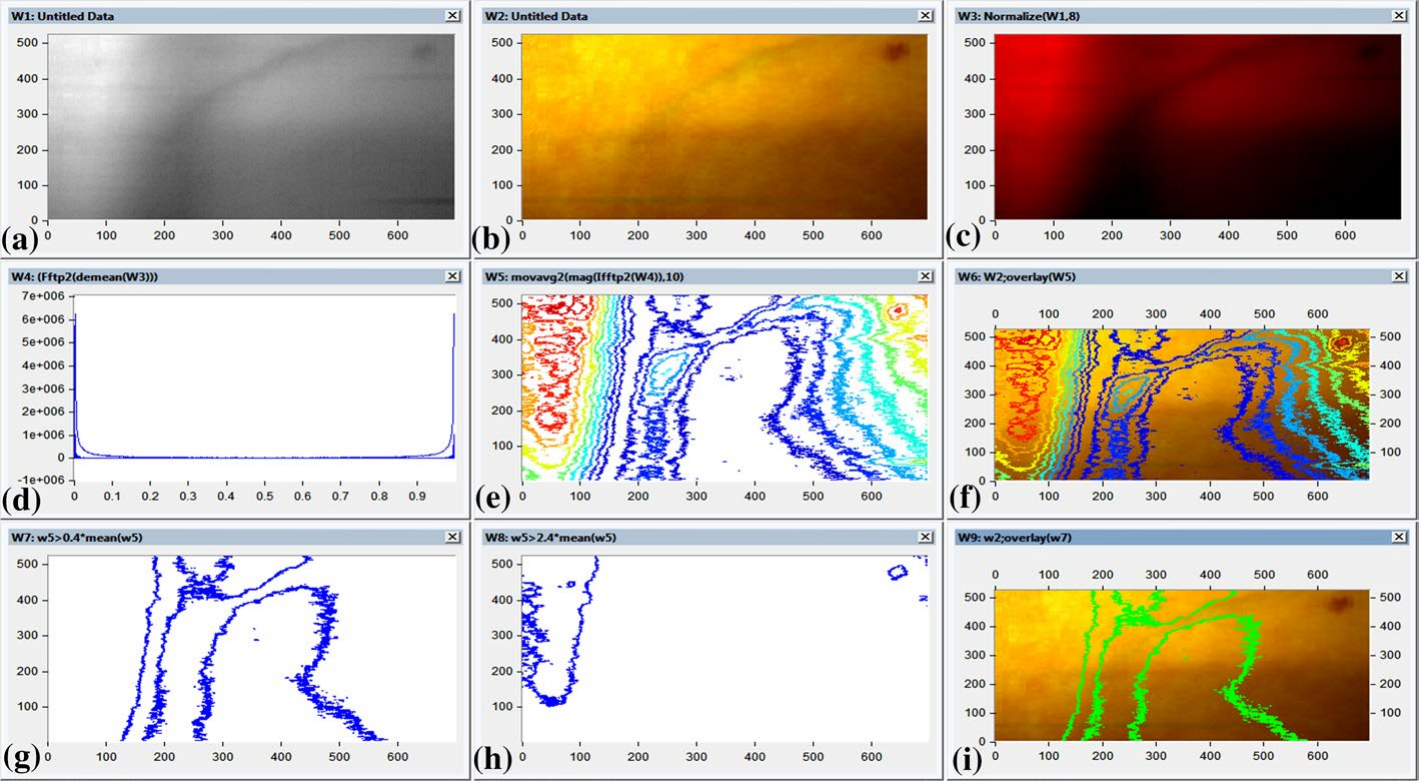
The Image processing of designed algorithm for discrimination between oxygenated and de-oxygenated blood oxygen concentration using 610 nm diffused; **a** The constructed spectral image for the patient forearm at wavelength 610 nm, **b** The segmented visible image over the band (400–700 nm), **c** The spectral image at wavelength 610 nm after image normalization, **d** Regarding the noise and DC removal using two-dimension fast Fourier transform for the spectral image at wavelength 610 nm, **e** The developed Time frequency domain image using two-dimensional inverse fast Fourier transform represented in contour map, **f** The contour mapping developed Time frequency domain image overlayed the segmented visible image over the band (400–700 nm) from Fig. 4b, **g** The normalized image from Fig. 4c after k-means segmentation to separate the vein position, **h** The normalized image from Fig. 4c after k-means segmentation to separate the atrium position, **i** The vein position image from Fig. 4g overlaying on the image of Fig. 4b

It is obvious that 610 nm is the optimum wavelength to discriminate between oxygenated and de-oxygenated blood concentration. Hyperspectral image at 610 nm was constructed, as represented in Fig. 4a. The segmented visible image over the band (400–700 nm) was constructed (Fig. 4b). Normalized hyperspectral image at 610 nm is represented (Fig. 4c). Noise and DC removal for normalized hyperspectral image at 610 nm was performed using two-dimension fast Fourier transform (Fig. 4d). Time frequency domain image was developed using two-dimensional inverse fast Fourier transform; the developed image was represented in contour map, as displayed in Fig. 4e. Contour map demonstrated high image contrast between oxygenated blood (red color) and deoxygenated blood (blue color) (Jones et al. 2001). Additionally contour map delineates high capability to locate atrium and vein location. In an attempt to evaluate the system capability to localize the vein and atrium location; the overlay of visible segmented image is shown in Fig. 4b. Contour map is displayed in Fig. 4e and f. In an attempt to separate and localize atrium or vein location; K-means analysis was performed on normalized hyperspectral image at 610 nm as represented in Fig. 4c. Vein and atrium locations are displayed in Fig. 4g and h, respectively. Overlay image of vein location on segmented visible image was constructed in Fig. 4i.

### Statistical analysis of diffused reflectance hyperspectral images

**Fig. 5:**
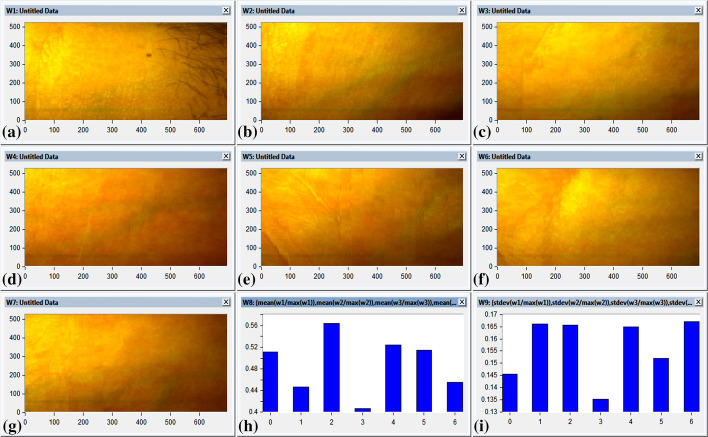
The Statistical analysis (mean and standard deviation) of segmented visible image over the band (400–700 nm) for the selected seven patients; **a** The segmented visible image over the band (400–700 nm) for the volunteer`s forearm Volunteer#1, **b** The segmented visible image over the band (400–700 nm) for the volunteer`s forearm Volunteer#2, **c** The segmented visible image over the band (400–700 nm) for the volunteer`s forearm Volunteer#3, **d** The segmented visible image over the band (400–700 nm) for the volunteer`s forearm Volunteer#4, **e** The segmented visible image over the band (400–700 nm) for the volunteer`s forearm Volunteer#5,**f** The segmented visible image over the band (400–700 nm) for the volunteer`s forearm Volunteer#6, **g** The segmented visible image over the band (400–700 nm) for the volunteer`s forearm Volunteer#7, **h** The statistical analysis exploiting the mean average technique for the spectral images of the selected seven patients, **i** The statistical analysis exploiting the standard deviation values for the spectral images of the selected seven patients

**Fig. 6:**
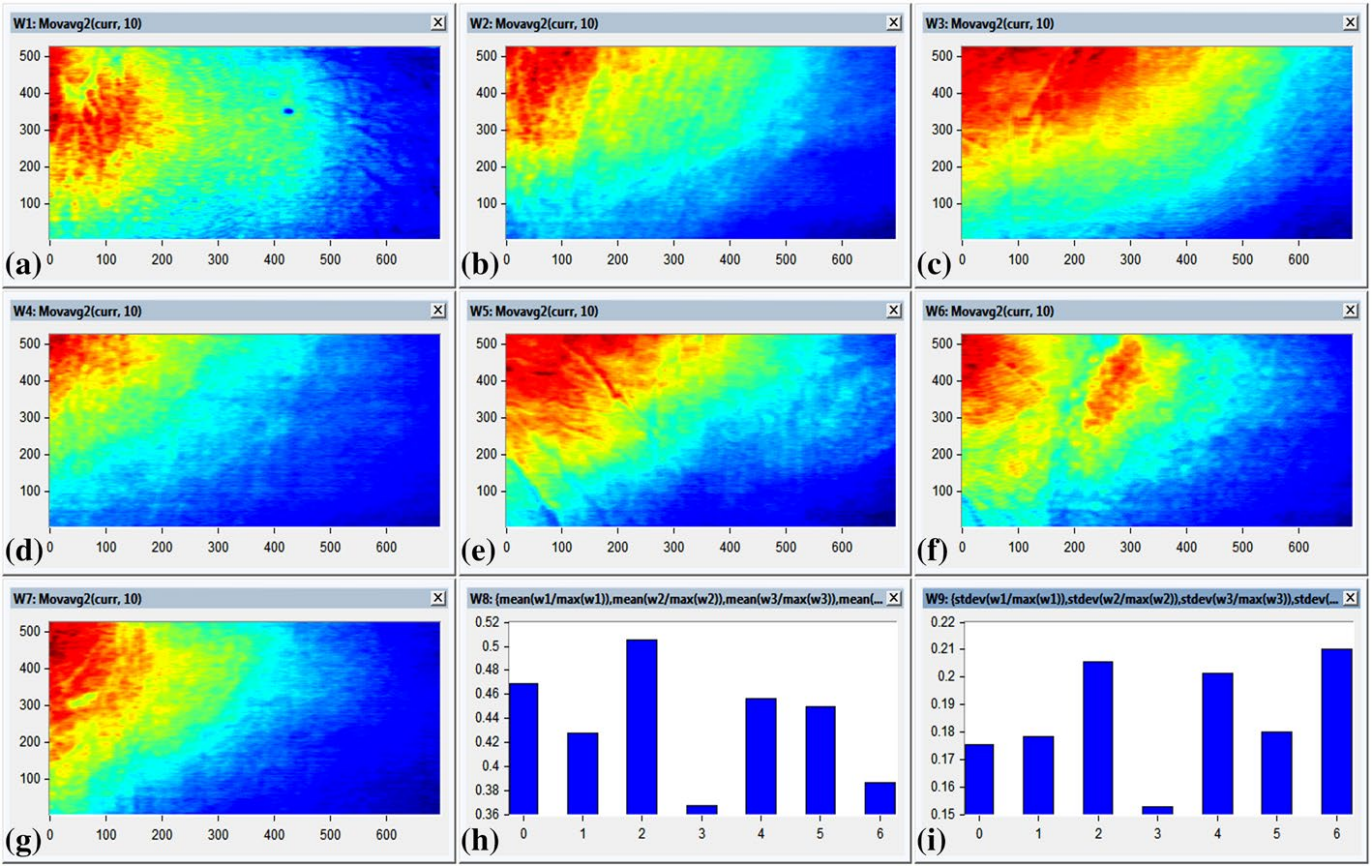
The captured spectral image at wavelength 610 nm after processed using normalized and frequency domain analysis for noise removal of the selected seven patients; **a** The captured spectral image at wavelength 610 nm after image processing for the volunteer`s forearm Volunteer#1, **b** The captured spectral image at wavelength 610 nm after image processing for the volunteer`s forearm Volunteer#2, **c** The captured spectral image at wavelength 610 nm after image processing for the volunteer`s forearm Volunteer#3, **d** The captured spectral image at wavelength 610 nm after image processing for the volunteer`s forearm Volunteer#4, **e** The captured spectral image at wavelength 610 nm after image processing for the volunteer`s forearm Volunteer#5, **f** The captured spectral image at wavelength 610 nm after image processing for the volunteer`s forearm Volunteer#6, **g** The captured spectral image at wavelength 610 nm after image processing for the volunteer`s forearm Volunteer#7, **h** The statistical analysis exploiting the mean average technique for the spectral images of the selected seven patients, **i** The statistical analysis exploiting the standard deviation values for the spectral images of the selected seven patients

To evaluate the capability of developing non-contact imaging system to measure the averaging between oxygenated and de-oxygenated blood was verified (Van Gastel et al. 2016a). We divide the investigated volunteers into two groups, group#1 (eight volunteers) and group#2 (seven volunteers). Group#1 were analyzed within the visible range (400–700 nm), as shown in Fig. 5. Where, statistical algorithm had been exploited using the mean value and standard deviation for each volunteer of the segmented diffused reflectance image to identify the optimum readings from the eight volunteers, as represented in Fig. 5h and 5i respectively. On the other side, group#2 were analyzed using the captured spectral images at wavelength 610 nm for the other seven volunteers. HS images had been processed using normalized and frequency domain analysis for noise removal for the seven patients, as displayed in Fig. 6. Then, the statistical analysis including mean and standard deviation values were represented in Fig. 6h, and 6i respectively. The estimated oxygen blood level for all volunteers was performed using non-contact imaging system versus the standard pulse oximeter.

Even though there was good agreement between statistical measurements of blood concentration to standard pulse oximeter; The measured values for group#1 of the volunteer`s scanned images over the visible band (400–700 nm) were represented associated with the mean and standard deviation values in Table 1, showing that there was variation in the mean value for the same oxygen blood level. For instance, different estimated oxygen blood level values were observed for the same oxygen blood levels of 90% using pulse oximeter.

**Table 1:** The scanned images of group#1 over the visible band (400–700 nm) highlighting the statistical analysis (mean, standard deviation, and Standard error) for each patient to identify the optimum reading versus the standard pulse oximeter measurements

No.	Patient ID	Reading	SPO_2_ measurements	Statistical analysis for the SPO_2_ measurements			Statistical analysis for the diffused reflectance image	
				Mean	Standard deviation	Standard error	Mean	Standard deviation
1	P#1	R#1	97	96.4	0.89	0.40	0.511	0.145
2		R#2	96					
3		R#3	97					
4		R#4	95					
5		R#5	97					
6	P#2	R#1	96	95.6	0.55	0.24	0.447	0.166
7		R#2	95					
8		R#3	96					
9		R#4	96					
10		R#5	95					
11	P#3	R#1	99	98.6	0.55	0.24	0.564	0.165
12		R#2	99					
13		R#3	98					
14		R#4	98					
15		R#5	99					
16	P#4	R#1	90	90.6	0.89	0.40	0.406	0.135
17		R#2	91					
18		R#3	92					
19		R#4	90					
20		R#5	90					
21	P#5	R#1	97	96.6	0.55	0.24	0.524	0.164
22		R#2	96					
23		R#3	97					
24		R#4	97					
25		R#5	96					
26	P#6	R#1	97	96.4	0.89	0.40	0.514	0.152
27		R#2	95					
28		R#3	96					
29		R#4	97					
30		R#5	97					
31	P#7	R#1	90	90.4	0.55	0.24	0.455	0.167
32		R#2	91					
33		R#3	91					
34		R#4	90					
35		R#5	90					
36	P#8	R#1	97	96.4	0.89	0.40	0.514	0.152
37		R#2	95					
38		R#3	96					
39		R#4	97					
40		R#5	97					

More representative statistical data are expected at enhanced spectral diffused reflectance image at 610 nm (Table 2). The statistical results for diffused reflectance images (mean and standard deviation values) were calculated and compared to standard pulse oximeter values. It is apparent that mean value was found to be in good agreement with pulse oximeter measurements, as represented in Table 2.

**Table 2:** The scanned images of group#2 at wavelength 610 nm after processed using normalized and frequency domain analysis for noise removal and highlighting the statistical analysis (mean, standard deviation, and Standard error) for each patient to identify the optimum reading versus the standard pulse oximeter measurements

No.	Patient ID	Reading	SPO2 measurements	Statistical analysis for the SPO_2_ measurements			Statistical analysis for the diffused reflectance image	
				Mean	Standard deviation	Mean	Mean	Standard deviation
1	P#1	R#1	97	96.6	0.55	0.24	0.469	0.175
2		R#2	97					
3		R#3	96					
4		R#4	96					
5		R#5	97					
6	P#2	R#1	96	95.8	0.45	0.20	0.427	0.178
7		R#2	96					
8		R#3	96					
9		R#4	96					
10		R#5	95					
11	P#3	R#1	98	98.6	0.55	0.24	0.505	0.205
12		R#2	99					
13		R#3	99					
14		R#4	98					
15		R#5	99					
16	P#4	R#1	90	90.6	0.89	0.40	0.367	0.152
17		R#2	91					
18		R#3	92					
19		R#4	90					
20		R#5	90					
21	P#5	R#1	97	96.6	0.55	0.24	0.456	0.201
22		R#2	96					
23		R#3	97					
24		R#4	97					
25		R#5	96					
26	P#6	R#1	97	96.8	0.45	0.20	0.450	0.180
27		R#2	97					
28		R#3	96					
29		R#4	97					
30		R#5	97					
31	P#7	R#1	90	90.4	0.55	0.24	0.381	0.210
32		R#2	91					
33		R#3	91					
34		R#4	90					
35		R#5	90					

## Conclusion

Maximum deviation in diffused reflectance according to SpO_2_ level took place at 610 nm. More precise statistical data were accomplished at enhanced spectral diffused reflectance image at 610 nm. The captured spectral images at 610 nm were processed using normalized and frequency domain analysis for noise removal. Diffused reflectance with HSI is a prospective technique to assist with phlebotomy and vascular approach. Additionally, it could permit future surgical or pharmacological intercessions that titrate or limit ischemic injury continuously. Furthermore, this technique could offer fast reliable indication of SpO_2_ levels for COVID-19 diagnosis. The presented results of the proposed prospective system could provide precise information (real-time, non-invasive) method. This innovation may likewise broaden the advantages of cutting-edge monitoring to more extensive patient populaces to assist with phlebotomy and vascular approach. Additionally, it may permit future surgical or pharmacological intercessions, and rapid diagnosis of COVID-19 pandemic.

## Data Availability

The authors stated and declare that all data is exist and available.

## Code availability

The authors stated and declare that all code is exist and available.
